# Propofol induces nuclear localization of Nrf2 under conditions of oxidative stress in cardiac H9c2 cells

**DOI:** 10.1371/journal.pone.0196191

**Published:** 2018-04-24

**Authors:** Takeaki Shinjo, Tatsuhide Tanaka, Hiroaki Okuda, Akira T. Kawaguchi, Kentaro Oh-hashi, Yuki Terada, Ayami Isonishi, Shoko Morita-Takemura, Kouko Tatsumi, Masahiko Kawaguchi, Akio Wanaka

**Affiliations:** 1 Department of Anesthesiology, Nara Medical University, Kashihara, Nara, Japan; 2 Department of Anatomy and Neuroscience, Nara Medical University, Kashihara, Nara, Japan; 3 Department of Functional Anatomy, Graduate School of Medical Science, Kanazawa University, Kanazawa, Ishikawa, Japan; 4 Department of Cell Transplantation and Regenerative Medicine, Tokai University School of Medicine, Isehara, Kanagawa, Japan; 5 Department of Chemistry and Biomolecular Science, Faculty of Engineering, Gifu University, Gifu, Japan; 6 United Graduate School of Drug Discovery and Medical Information Sciences, Gifu University, Gifu, Japan; National Institutes of Health, UNITED STATES

## Abstract

Oxidative stress contributes to myocardial ischemia-reperfusion injury, which causes cardiomyocyte death and precipitate life-threatening heart failure. Propofol has been proposed to protect cells or tissues against oxidative stress. However, the mechanisms underlying its beneficial effects are not fully elucidated. In the present study, we employed an *in vitro* oxidative injury model, in which rat cardiac H9c2 cells were treated with H_2_O_2_, and investigated roles of propofol against oxidative stress. Propofol treatment reduced H_2_O_2_-induced apoptotic cell death. While H_2_O_2_ induced expression of the antioxidant enzyme HO-1, propofol further increased HO-1 mRNA and protein levels. Propofol also promoted nuclear localization of Nrf2 in the presence of H_2_O_2_. Knockdown of Nrf2 using siRNA suppressed propofol-inducible Nrf2 and expression of Nrf2-downstream antioxidant enzyme. Knockdown of Nrf2 suppressed the propofol-induced cytoprotection. In addition, Nrf2 overexpression induced nuclear localization of Nrf2 and HO-1 expression. These results suggest that propofol exerts antioxidative effects by inducing nuclear localization of Nrf2 and expression of its downstream enzyme in cardiac cells. Finally, we examined the effect of propofol on cardiomyocytes using myocardial ischemia-reperfusion injury models. The expression level of Nrf2 protein was increased at 15 min after reperfusion in the ischemia-reperfusion and propofol group compared with ischemia-reperfusion group in penumbra region. These results suggest that propofol protects cells or tissues from oxidative stress via Nrf2/HO-1 cascade.

## Introduction

Oxidative stress contributes to many pathological conditions, including tissue ischemia, neurological disorders, cancer, hypertension, atherosclerosis, diabetes, idiopathic pulmonary fibrosis and asthma [1]. Oxidative stress causes an overabundance of oxidants, such as reactive oxygen species (ROS), that are highly reactive and can damage cell components, including carbohydrates, lipids, nucleic acids and proteins, and alter their functions [1]. In the case of cardiac diseases, oxidative stress plays a major role in myocardial ischemia-reperfusion injury that results in cardiac cell death and subsequent heart failure [2].

Propofol (2, 6-diisopropylphenol) is used to sedate patients during surgery [3]. The anesthetic effect of propofol has been attributed to activation of GABA A receptors, and consequent slowing of the channel-closing time. Propofol also acts as a sodium channel blocker [4]. In addition to its anesthetic effects, propofol reportedly protects cells or tissues from oxidative stress [5, 6]. The underlying mechanisms of this beneficial effect have not been elucidated. In some cases, however, propofol showed cytotoxic effects [7, 8]. Tsuchiya et al. [9] demonstrated that propofol could induce apoptosis in cultured human promyelocytic leukemia HL-60 cells via activation of the cell surface death receptor pathway and the mitochondrial pathway. These discrepancies may be attributed to differences in cell types and/or in experimental paradigms. Whether propofol has beneficial or harmful effects on particular cell types or tissues is clinically important, since propofol is commonly used in surgery, in which the human body receives invasive stress.

Heme oxygenase-1 (HO-1) is an antioxidant enzyme that can be induced by oxidative stress [10]. It catalyzes the rate-limiting step in heme degradation, leading to generation of equimolar amounts of iron ions, biliverdin and CO [10]. Cardiac-specific HO-1 overexpression protects against myocardial ischemia and reperfusion injury [11] and improves cardiac function in an animal model [12]. HO-1 expression is regulated by NF-E2-related factor 2 (Nrf2), a transcription factor that is responsible for the regulation of cellular redox balance [10]. It has been reported that Nrf2 is the principal transcription factor that regulates antioxidant response element-mediated expression of antioxidant enzymes [13, 14]. Hao et al. reported that Nrf2 is a key molecule that inhibited endotoxin-induced myocardial toxicity using a mouse model [15]. Although the activation of Nrf2/HO-1 by propofol has been reported in a rat liver transplantation model [5, 16], little is known from cardiomyocyte models about the relationship between Nrf2/HO-1 cascades and propofol. In the present study, we employed a H_2_O_2_-induced oxidative stress model to investigate directly the role of propofol against ROS in rat cardiac H9c2 cells.

## Materials and methods

### Cell culture

H9c2 rat cardiac myoblast cells (American Type Culture Collection, Manassas, VA, CRL-1446) were cultured in Dulbecco's modified Eagle's medium (DMEM) supplemented with 10% fetal bovine serum, 100 U/ml penicillin and 100 μg/ml streptomycin. Cells were grown in a humidified incubator containing an atmosphere of 95% air / 5% CO_2_ at 37°C.

### Reagents for cell culture

Propofol and H_2_O_2_ were purchased from Wako Pure Chemical Industries (Osaka, Japan). Propofol was dissolved in dimethyl sulfoxide. H9c2 cells were incubated with 100 μM propofol for 30 min and then for a further 24 h after the addition of H_2_O_2_. Nrf2 siRNA, HO-1 siRNA and Nqo-1 siRNA (20 nM; stealth siRNA; Invitrogen, Stockholm, Sweden) or control siRNA (20 nM; stealth RNAi negative control; Invitrogen) were used to transfect into H9c2 with Lipofectamine 2000 (Invitrogen) according to the manufacturer’s protocol.

### Lactate dehydrogenase assay

Cytotoxicity to H9c2 cells was measured using a Cytotoxicity LDH Assay kit (Dojindo Molecular Technologies, Japan). H9c2 cells were seeded in 96-well cell culture plates at a density of 2× 10^4^ cells per well and cultured in medium containing 100, 500 or 1000 μM H_2_O_2_, or 10, 100 or 1000 μM propofol. The experimental assay was performed following the manufacturer’s protocol. LDH catalyzes the conversion of lactate to pyruvate upon reduction of NAD^+^ to NADH/H^+^; the added tetrazolium salt is reduced to formazan. The amount of formazan formed correlates with LDH activity. The formazan product was measured with a microtiter plate reader at 490 nm.

### TUNEL assay

H9c2 cells were seeded in 4-well cell culture plates at a density of 2× 10^4^ cells per well and cultured in medium containing 250 μM H_2_O_2_, or 100 μM propofol. Apoptotic cells were identified by terminal deoxynucleotidyl transferase-mediated dUTP nick end-labeling (TUNEL) staining using an In Situ Cell Death Detection Kit (Wako) according to the manufacturer's instructions. Briefly, H9c2 cells were fixed with 4% paraformaldehyde for 10 min at room temperature, washed twice with phosphate-buffered saline (PBS), permeabilized with 0.1% Triton X-100 in 0.1% sodium citrate and then rinsed with PBS. Cells were stained with 100 μl terminal deoxynucleotidyl transferase reaction solution at 37°C for 10 min, and then incubated with 3% H_2_O_2_ solution to eliminate intrinsic peroxidase activity. Apoptotically fragmented DNA was detected using horseradish peroxidase antibody and DAB as a substrate.

### Western blotting

H9c2 cells were seeded in 6-cm dishes at a density of 5× 10^5^ cells and cultured in medium containing 250 μM H_2_O_2_, or 100 μM propofol. Samples (cells or tissues) were lysed with 10 mM Tris, pH 7.4, containing 150 mM NaCl, 5 mM EDTA, 1% Triton X-100, 1% deoxycholic acid and 0.1% sodium dodecyl sulfate (SDS). The homogenate was centrifuged at 20,600 *g* for 5 min, and the supernatant was stored at -20°C. To prepare nuclear extracts, cells were lysed with 20 mM HEPES, pH 7.6, containing 10 mM NaCl, 0.2 mM EDTA, 1.5 mM MgCl_2_, 1 mM DTT, 0.1% NP-40 and 20% glycerol. The homogenate was centrifuged at 300 *g* for 5 min, and the pellet was lysed with 20 mM HEPES, pH 7.6, containing 500 mM NaCl, 0.2 mM EDTA, 1.5 mM MgCl_2_, 1 mM DTT, 0.1% NP-40 and 20% glycerol. The homogenate was centrifuged at 20,600 *g* for 15 min, and the supernatant was stored at -20°C. Protein concentration was measured using a bicinchoninic acid protein assay kit (Pierce). Equal amounts of protein per lane were electrophoresed on SDS-polyacrylamide gels, and then transferred to a polyvinylidene difluoride membrane. The blots were probed with anti-HO-1 (1:1000; Enzo Life Sciences, ADI-SPA-896, rabbit polyclonal), anti-Nrf2 (1:500; abcam, ab137550, rabbit polyclonal), anti-GAPDH (1:5000; Millipore, ABS16, rabbit polyclonal), anti-c-Myc (Wako, 017–2187, mouse monoclonal) or anti-Histone H1 (1:1000; Abcam, ab61177, rabbit polyclonal) antibodies. Immunoblot analysis was performed with horseradish peroxidase-conjugated anti-mouse and anti-rabbit IgG using enhanced chemiluminescence Western blotting detection reagents (Wako). Data were acquired in arbitrary densitometric units using Scion image software.

### RT-PCR

H9c2 cells were seeded in 6-well cell culture plates at a density of 1× 10^5^ cells per well and cultured in medium containing 250 μM H_2_O_2_, or 100 μM propofol. Total RNA of cells or tissues were extracted using a NucleoSpin RNA kit (Macherey-Nagel, Düren, Germany). Total RNA extracts were reverse-transcribed using random primers and a QuantiTect Reverse Transcription kit (QIAGEN, Hilden, Germany), according to the manufacturer’s instructions. Real-time PCR was performed using a LightCycler Quick System 350S (Roche Diagnostics), with THUNDERBIRD SYBR qPCR Mix (Toyobo, Osaka, Japan). PCR primers used in this study were as follows: GAPDH sense primer, 5’-CTACATGGTCTACCTGTTCCAG-3’; GAPDH antisense primer, 5’-AGTTGTCATGGATGACCTTGG-3’; HO-1 sense primer, 5’-AGCATGTCCCAGGATTTGTC-3’; HO-1 antisense primer, 5’-ACTGGGTTCTGCTTGTTTCG-3’; Nqo-1 sense primer, 5’-CGCAGAGAGGACATCATTCA-3’; Nqo-1 antisense primer, 5’-CGCCAGAGATGACTCAACAG-3’ Nrf2 sense primer, 5’-GCAACTCCAGAAGGAACAGG-3’; Nrf2 antisense primer, 5’-GGAATGTCTCTGCCAAAAGC-3’.

### Immunocytochemistry

H9c2 cells were seeded in 3.5-cm dishes at a density of 2× 10^3^ cells per well and cultured in medium containing 250 μM H_2_O_2_, or 100 μM propofol. Cultured cells were fixed with 4% paraformaldehyde for 1 h at 25°C and incubated with blocking solution containing 5% BSA and 0.1% Triton X-100 in PBS for 1 h. Primary anti-Nrf2 antibody (1:500; Santa Cruz Biotechnology, sc-722) was applied overnight at 4°C. Alexa Fluor 594-conjugated anti-rabbit IgG (1:1000, Life Technologies) or Alexa Fluor 488-conjugated anti-phalloidin (1:500; Invitrogen) was used as the secondary antibody.

### Generation of construct and transient transfection in H9c2 cells

PCR cloning was performed to amplify Nrf2 cDNA with a primer having an optimal Kozak consensus sequence just before the in-frame first ATG of rat Nrf2 gene. cDNA fragment was inserted into the pcDNA3.1/Myc-His vector (Invitrogen). By using the Lipofectamine 2000 (Invitrogen), H9c2 cells were transfected with a Kozak-Nrf2 construct according to the manufacturer’s instructions.

### *In vivo* myocardial ischemia/reperfusion model

All experiments were approved by the Institutional Review Board of Tokai University (#171026). The animals received humane care as required by the institutional guidelines for animal care and treatment in experimental investigations. Male Lewis rats (6–8 weeks) were randomly divided into two groups: 1) ischemia/reperfusion (I/R) group; 2) I/R+propofol group. All rats were anesthetized with 3% sevoflurane inhalation. Rectal temperature was monitored and maintained at 36.0°C by servo-controlled water pad. An indwelling catheter for infusion by syringe pump of each solution (saline, propofol) was inserted into the tail vein. A left thoracotomy was performed to expose the heart and sevoflurane was reduced to 2% to the end of experiment. The myocardial I/R model was induced by ligation with a 7–0 manofilament suture of the left anterior descending (LAD) coronary artery for 30 minutes followed by reperfusion for 15 min or 90 min. The rats in the I/R group received an intravenous infusion of physiologic saline (3 ml/hr) for 45 min or 120 min. In the I/R+propofol group, 22 mg/kg/hr propofol was started 10 min after LAD occlusion and administered for 35 min or 110 min. At the end of the ischemic period, the suture was released to induce reperfusion of the myocardium for 15 min or 90 min. The following territories of the heart were investigated: 1) Infarction: tissue most vulnerable to damage during a LAD coronary artery occlusion were located at the vascular bed of the LAD. 2) Penumbra: tissue within the vascular bed of the LAD comprised the area at risk.

### Statistical analysis

Quantifications were performed from at least three independent experimental groups. Data are presented as mean ± SEM. Statistical analyses were performed using Student’s *t*-test or Welch’s *t*-test for two groups or one-way ANOVA for multiple groups, and significant differences between group means were identified with the Tukey–Kramer test. Statistical significance is indicated as asterisks. *P < 0.05, **P < 0.01. All n and *P* values are indicated in figure legends.

## Results

### Effects of H_2_O_2_ and propofol on cell viability

**Fig 1:**
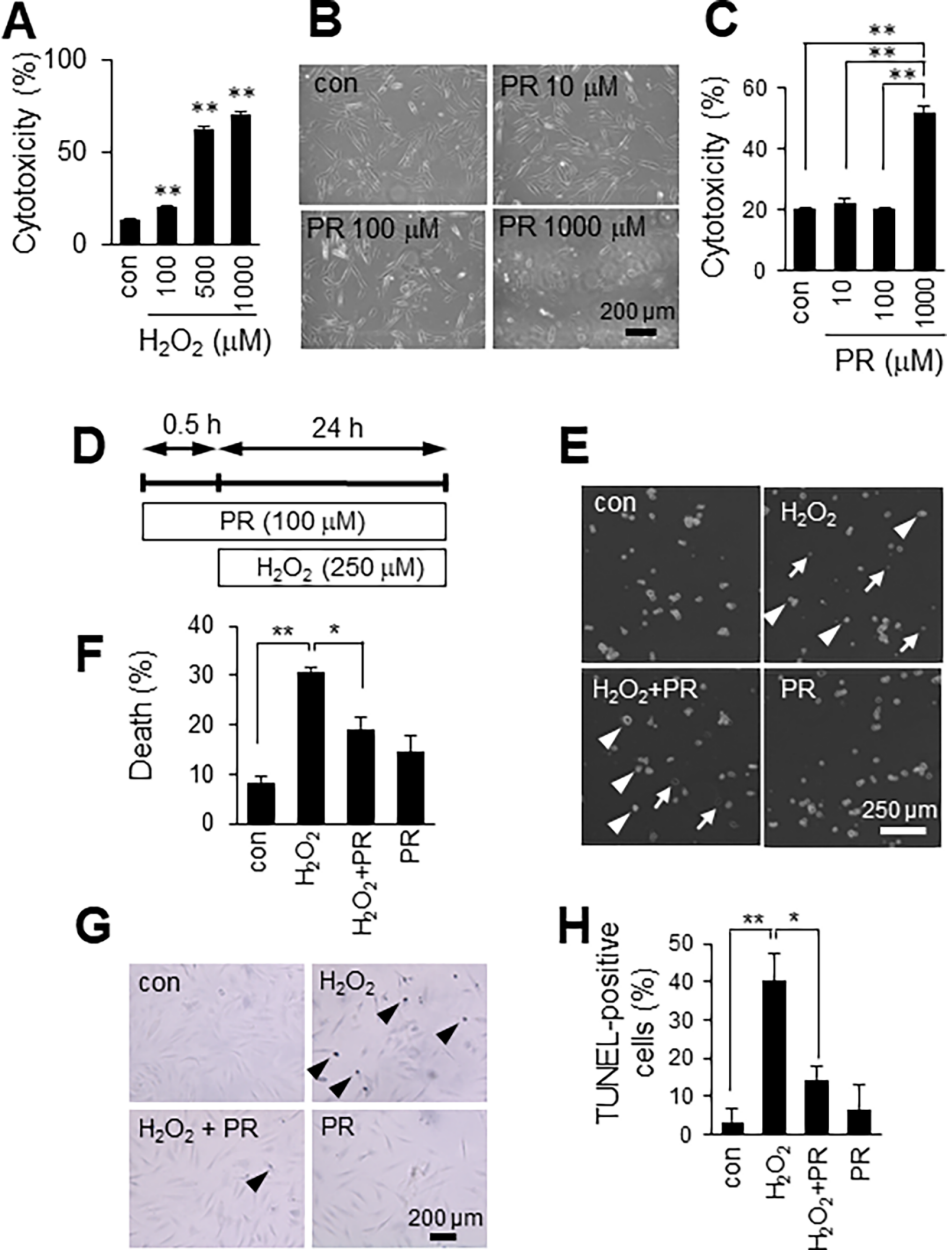
Propofol inhibits H_2_O_2_-induced H9c2 cell death. **A**: Effect of H_2_O_2_ on cell viability. Cytotoxicity was measured using an LDH assay. n = 3. **B**: Effect of propofol (PR) on cell viability. Scale bar, 200 μm. **C**: Quantitative analysis of PR-induced cytotoxicity in H9c2 cells. n = 3. **D**: Diagram of the scheme of propofol (PR) and H_2_O_2_ treatment. **E**: Effect of PR on cell viability under oxidative stress conditions. Arrows, dead cells; arrowheads, surviving cells. Scale bar, 250 μm. **F**: Quantification of dead H9c2 cells. n = 3. The data are expressed as mean ± SEM. **G**: TUNEL staining. Arrowheads, TUNEL-positive cells. Scale bar, 200 μm. **H**: Quantification of TUNEL-positive cells. n = 3. The data are expressed as mean ± SEM. Control (con) means no treatment. *P < 0.05, **P < 0.01.

We first determined the dose at which cytotoxicity develops after 24 h H_2_O_2_ exposure in H9c2 cells. As shown in Fig 1A, H_2_O_2_ caused cell death in a concentration-dependent manner over the tested range (100 to 1000 μM). Propofol has been proposed to protect cells or tissues from oxidative stress [5, 6], but reportedly shows cytotoxic effects in cells from the immune system [7, 8]. Therefore, we next tested whether propofol was cytotoxic to H9c2 cells. We determined cell cytotoxicity after treatment with propofol (10 to 1000 μM) for 24 h, using phase-contrast microscopy and an LDH assay. Cell viability was not affected by propofol at 10 or 100 μM, but cell death was observed with 1000 μM propofol (Fig 1B and 1C). Because the aim of this study was to investigate protective roles of propofol against ROS, we used 250 μM H_2_O_2_, 100 μM propofol for subsequent experiments.

### Propofol inhibits H_2_O_2_-induced H9c2 cell death

To determine the effects of propofol on H_2_O_2_-induced cell death, H9c2 cells were pretreated with propofol (100 μM) for 30 min, and then co-incubated with 250 μM H_2_O_2_ for an additional 24 h (Fig 1D). We checked the protective effects of propofol on H_2_O_2_-induced cell death by a trypan blue exclusion test. As shown in Fig 1E and 1F, propofol reduced H_2_O_2_-induced cell death (H_2_O_2,_ 30.7 ± 1.1%; H_2_O_2_ + PR, 19.1 ± 2.4%). TUNEL staining revealed that 250 μM H_2_O_2_ induced apoptosis in H9c2 cells (40.2 ± 7.3%) and that 100 μM propofol reduced H_2_O_2_-induced apoptosis (14.4 ± 3.5%) (Fig 1G and 1H).

### Propofol and H_2_O_2_ synergistically increase HO-1 expression in H9c2 cells

**Fig 2:**
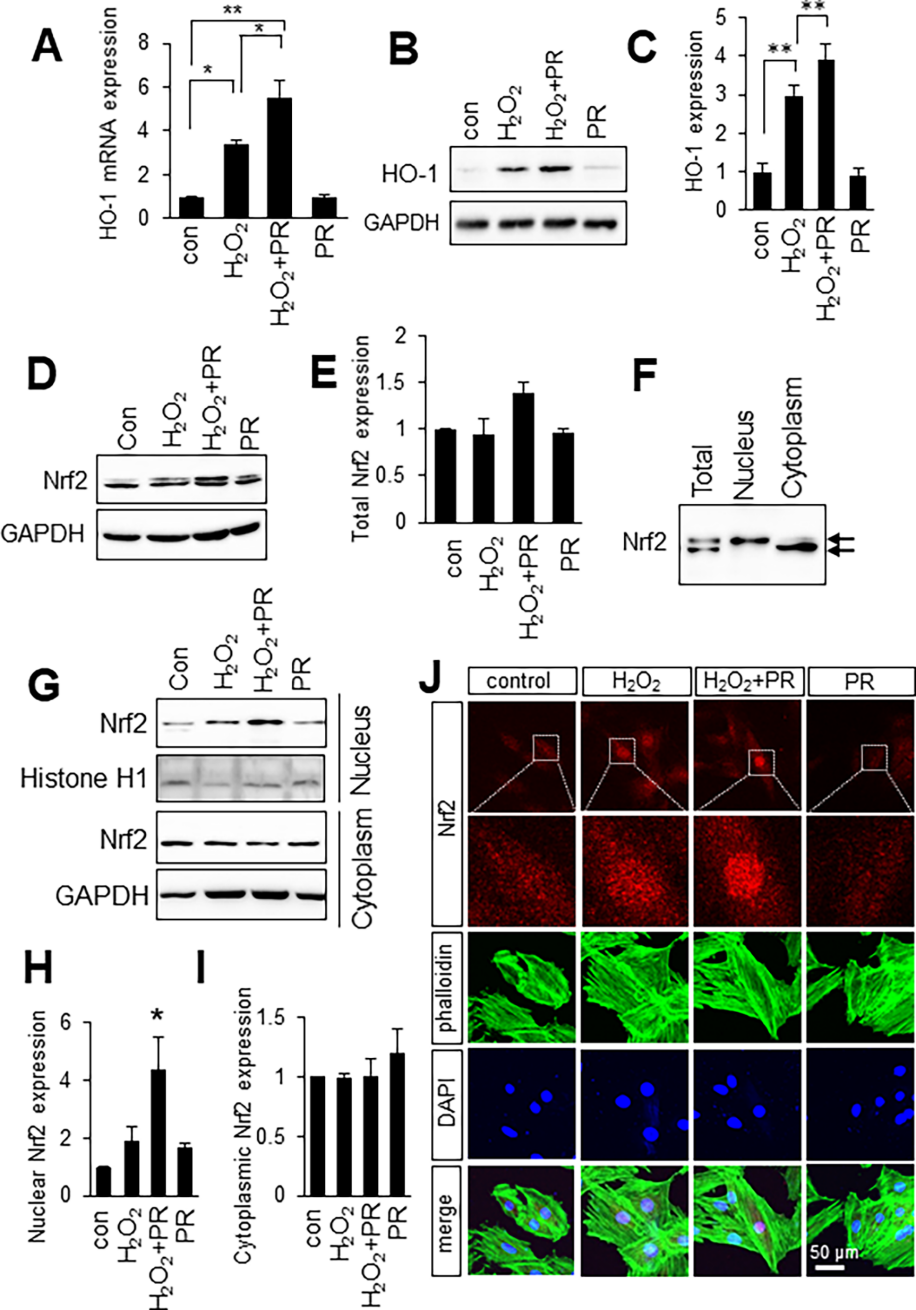
Propofol upregulate HO-1 expression and nuclear localization of Nrf2 under oxidative stress conditions. **A**: mRNA expression profile for HO-1. n = 3. The data are expressed as mean ± SEM. **B**: Western blot analysis of HO-1 in H9c2 cells. **C**: Quantification of the density of expression level of HO-1. n = 3. The data are expressed as mean ± SEM. **D**: Western blot analysis of Nrf2 in H9c2 cells. **E**: Quantification of the density of expression level of total Nrf2. n = 3. The data are expressed as mean ± SEM. **F**: Western blot analysis of Nrf2 in total lysates, nuclear lysates and cytoplasmic lysates of H9c2 cells. **G**: Western blot analysis of Nrf2 in nuclear lysates and cytoplasmic lysates of H9c2 cells. **H**, **I**: Quantification of the density of expression level of nuclear Nrf2 and cytoplasmic Nrf2. **J**: Representative immunocytochemical labeling of Nrf2 in H9c2 cells treated with H_2_O_2_ and propofol (PR). Scale bar, 50 μm. Control (con) means no treatment. *P < 0.05, **P < 0.01.

We next examined the expression profiles of oxidative stress-related factors. HO-1 is an important component of the cellular defense mechanism against oxidative stress [10]. Thus, we determined whether propofol induced HO-1 expression in H9c2 cells. As shown in Fig 2A, H_2_O_2_ significantly increased the expression of HO-1 mRNA (3.4-fold of control). Interestingly, propofol showed a synergistic effect on HO-1 mRNA expression level in H_2_O_2_-treated H9c2 cells (5.5-fold of control), whereas propofol treatment alone did not increase HO-1 mRNA expression (0.95-fold of control). HO-1 protein expression in H9c2 cells was consistent with its mRNA expression patterns (Fig 2B, S1A and S1B Fig). H_2_O_2_ induced HO-1 protein (3.0-fold of control) and propofol further increased HO-1 protein level (3.9-fold of control). These results indicate that propofol increased HO-1 expression specifically under oxidative stress.

### Propofol upregulate nuclear localization of Nrf2

It has been reported that antioxidant genes including HO-1 are regulated by the transcription factor Nrf2 [10]. We next investigated whether propofol-induced HO-1 expression is Nrf2-dependent in H9c2 cells. The Nrf2 level in total lysates of H9c2 cells was higher in cells pretreated with 100 μM propofol for 30 min and then incubated with 250 μM H_2_O_2_ than in cells treated with only 250 μM H_2_O_2_ (Fig 2D and 2E, S1C and S1D Fig), although the difference did not reach statistical significance. We noticed that Nrf2 was detected as two bands around 110 kDa. We prepared nuclear extracts and cytoplasmic extracts of H9c2 cells and performed Western blotting. Western blot analysis detected molecular weight of nuclear Nrf2 was higher than that of cytoplasmic Nrf2 (Fig 2F, S1E Fig). It has been reported that under normal conditions, Nrf2 and Keap1 are complexed in the cytoplasm where they are targeted for degradation. On the other hand, Nrf2 is released from Keap1 and translocates to the nucleus [17]. We next investigated whether propofol-induced nuclear Nrf2 expression in H9c2 cells, using Histone H1 as an internal control for nuclear proteins. As shown in Fig 2G and 2H, propofol significantly increased the expression level of nuclear Nrf2 (4.4-fold of control) under conditions of oxidative stress in H9c2 cells (S1F and S1G Fig), whereas cytoplasmic Nrf2 was not changed (Fig 2G and 2I, S1H and S1I Fig). Notably, combined propofol and H_2_O_2_ treatment induced nuclear localization of Nrf2, whereas single propofol treatment did not (Fig 2G and 2H, S1F and S1G Fig). Immunocytochemistry also demonstrated that propofol promoted nuclear localization of Nrf2 in the presence of H_2_O_2_ (Fig 2J). These results suggest that propofol induced HO-1 expression by promoting nuclear localization of Nrf2 under oxidative stress conditions.

### Nrf2 inhibition suppresses propofol-induced antioxidant expression and cytoprotection

**Fig 3:**
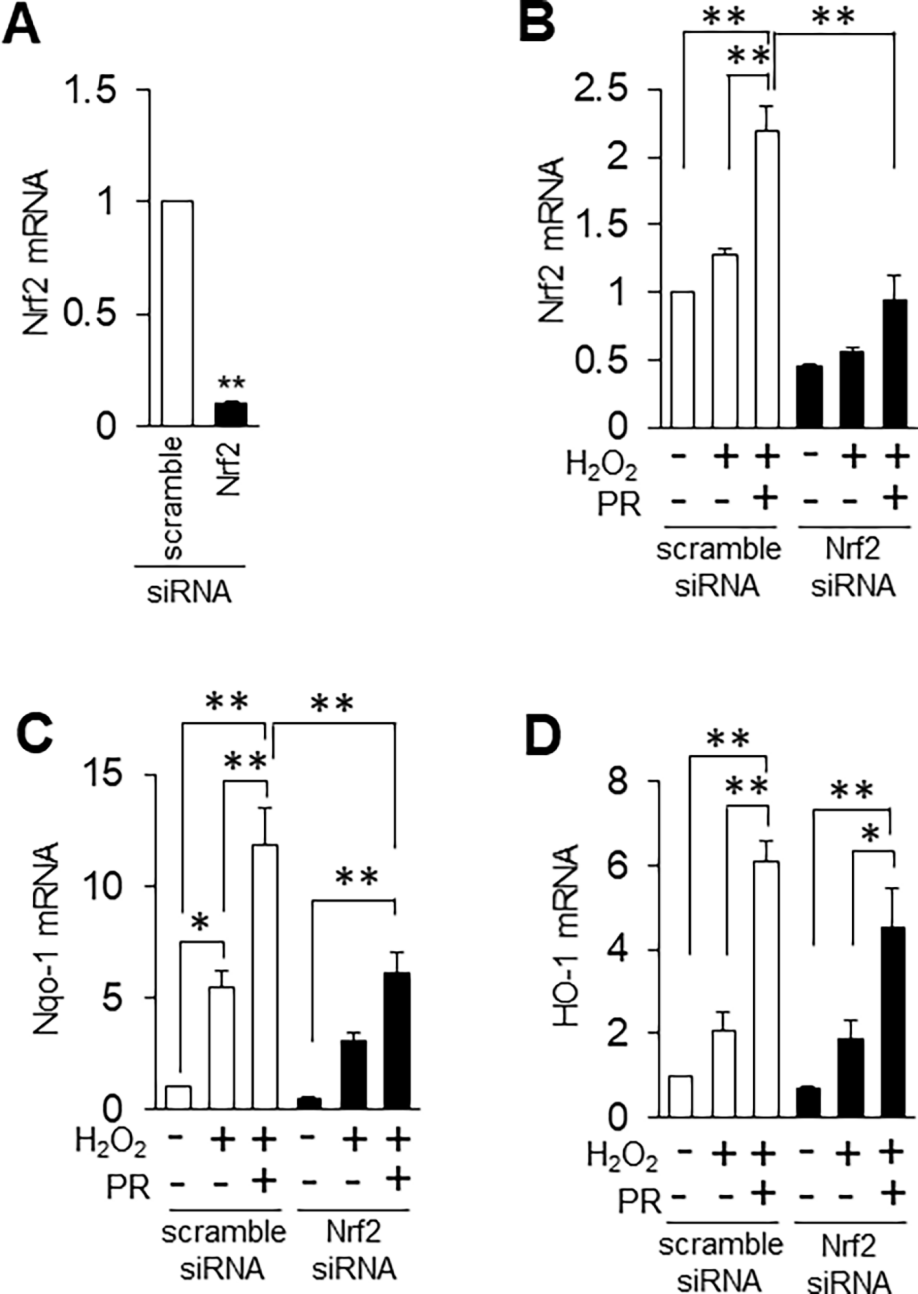
Knockdown of Nrf2 by siRNA suppresses propofol-induced antioxidant expression. **A**: Expression of Nrf2 mRNA in H9c2 cells transfected with Nrf2 and scramble (control) siRNA. n = 3. The data are expressed as mean ± SEM. **B-D**: Expression profiles of Nrf2 (B), HO-1 (C) and Nqo-1 (D) mRNAs in H9c2 cells transfected with scramble, Nrf2 and HO-1 siRNA. n = 3. The data are expressed as mean ± SEM. *P < 0.05, **P < 0.01.

**Fig 4:**
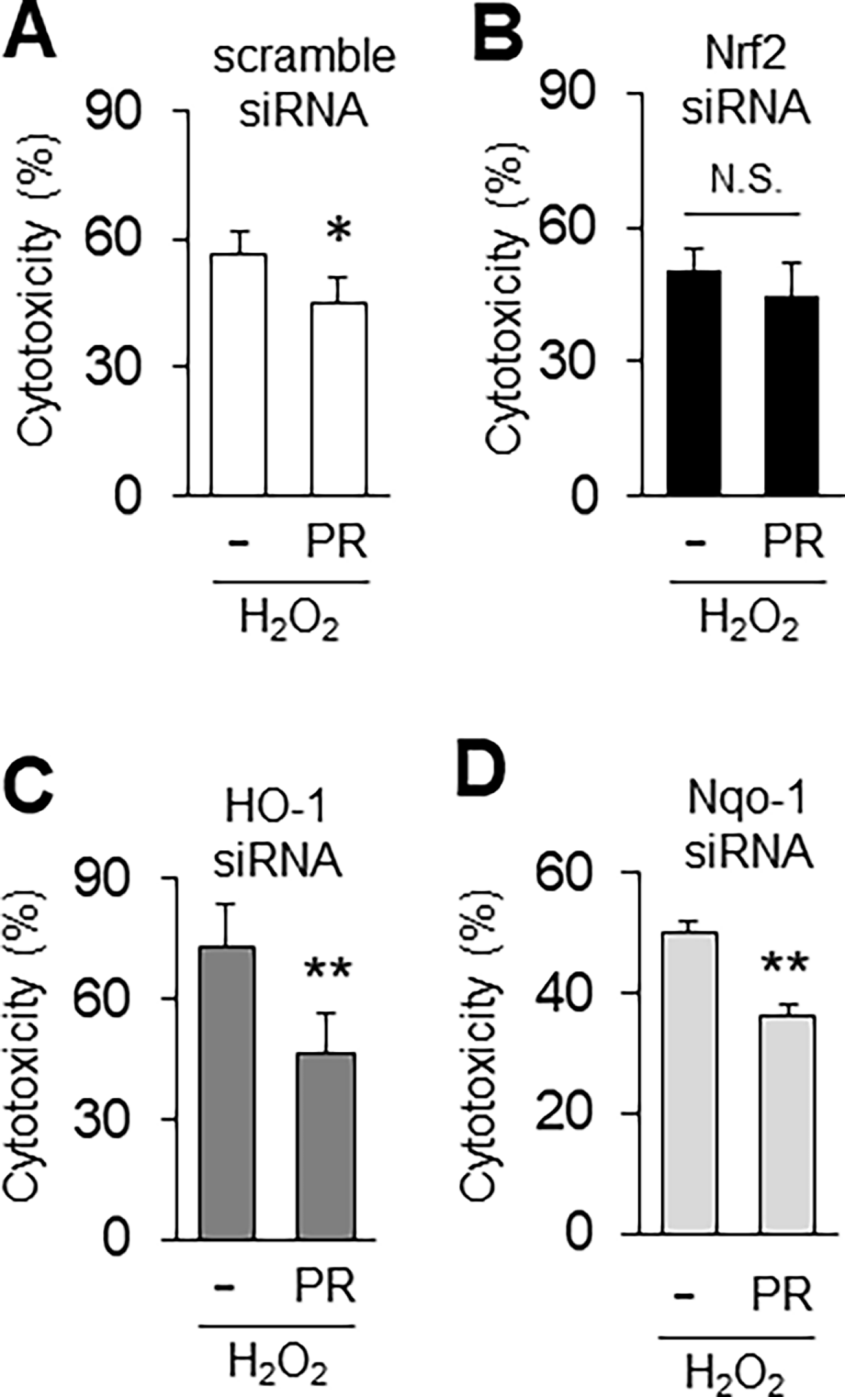
Nrf2 inhibition suppresses propofol-induced cytoprotection. **A-D**: Effect of Nrf2, HO-1, Nqo-1 or scramble siRNA on propofol-induced cytoprotection, after at 24 h after treatment with H_2_O_2_. Cytotoxicity was measured using an LDH assay. n = 3. The data are expressed as mean ± SEM. *P < 0.05, **P < 0.01. N.S., not significant.

We found that nuclear localization of Nrf2 was increased by propofol under oxidative stress conditions (Fig 2). Therefore, we examined whether reducing Nrf2 expression using siRNA affects antioxidants. As a preliminary experiment, we transfected siRNA against Nrf2 into H9c2 cells, and measured Nrf2 mRNA level by qRT-PCR. We found that Nrf2 siRNA strongly suppressed Nrf2 mRNA (9.9% of control siRNA) (Fig 3A). We next examined the expression of propofol-induced antioxidants in the presence of siRNA under oxidative stress conditions. Nrf2 siRNA inhibited propofol-inducible Nrf2 mRNA expression (Fig 3B). Nrf2 activates transcription of many cytoprotective enzymes, and Nqo-1 is an additional representative antioxidant enzyme whose expression is regulated by Nrf2 [18]. Nqo-1 mRNA was elevated by propofol under oxidative stress conditions, and its mRNA was significantly reduced by Nrf2 siRNA (Fig 3C). HO-1 mRNA was also slightly, albeit not significantly, reduced by Nrf2 siRNA under oxidative stress conditions in the presence of propofol (Fig 3D). These results suggest that propofol-induced Nrf2 expression is important for antioxidant gene expression. We next asked whether Nrf2 siRNA can suppress the propofol-induced cytoprotection in H9c2 cells. We determined cytotoxicity using the LDH assay after transfection of siRNA against Nrf2. At 24 h after treatment with H_2_O_2_, propofol reduced the H_2_O_2_-induced cytotoxicity of H9c2 cells transfected with scramble (control) siRNA (H_2_O_2_, 56.5% ± 5.3%; H_2_O_2_+PR, 45.2% ± 6.9%) (Fig 4A). On the other hand, propofol did not reduce the H_2_O_2_-induced cytotoxicity of H9c2 cells transfected with Nrf2 siRNA (H_2_O_2_, 50.6% ± 4.8%; H_2_O_2_+PR, 44.5% ± 7.6%) (Fig 4B). HO-1 siRNA and Nqo-1 siRNA did not have these effects, that is, propofol reduced the H_2_O_2_-induced cytotoxicity of H9c2 cells transfected with HO-1 and Nqo-1 siRNA (HO-1 siRNA: H_2_O_2_, 72.7% ± 10.8%; H_2_O_2_+PR, 46.6% ± 9.7%, Nqo-1 siRNA: H_2_O_2_, 50.1% ± 1.6%; H_2_O_2_+PR, 36.4% ± 1.7%) (Fig 4C and 4D). These results suggest that propofol-induced Nrf2 is critical for cytoprotection against oxidative stress.

### Nrf2 overexpression induce nuclear localization of Nrf2 and HO-1 expression

**Fig 5:**
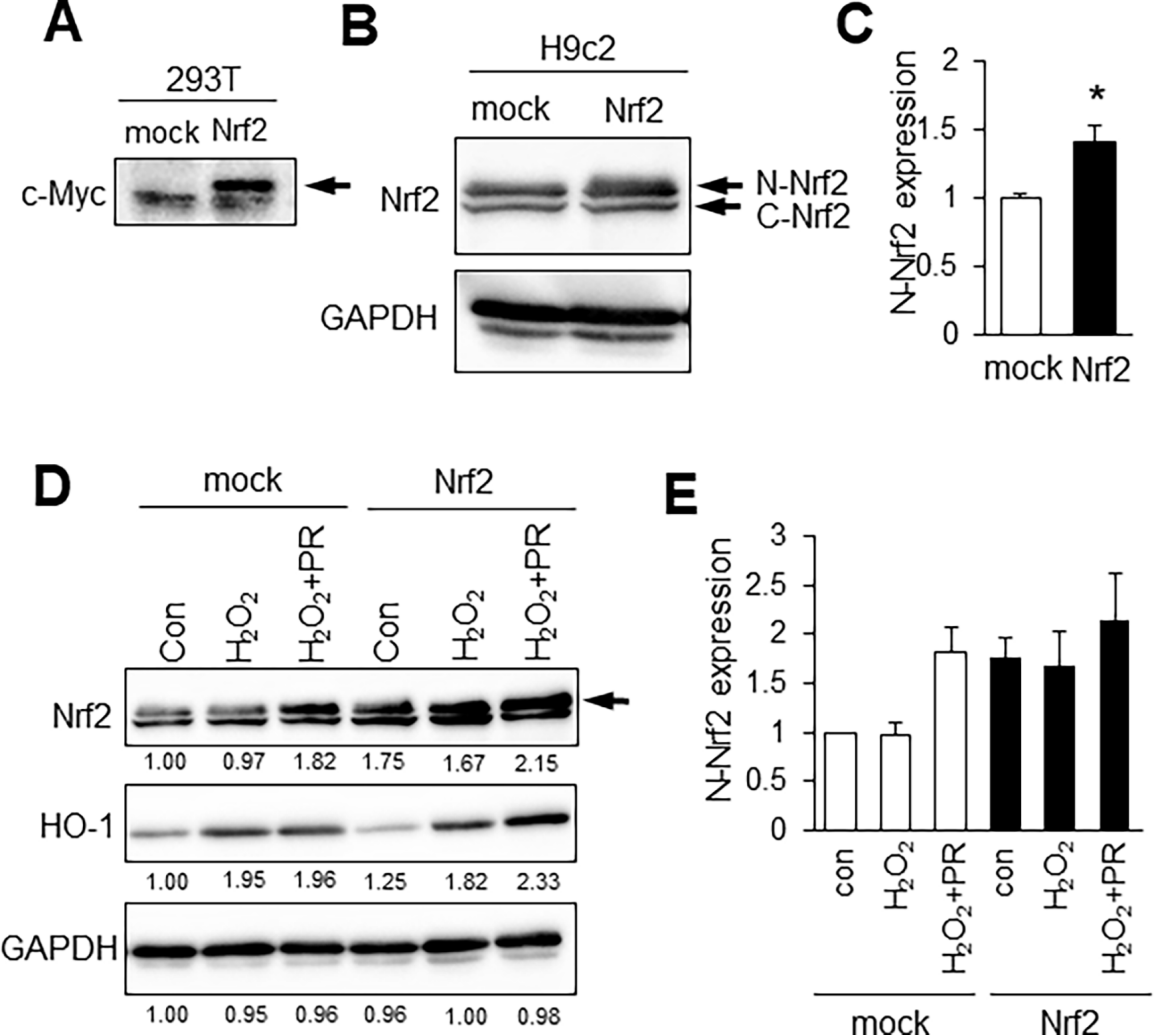
Nrf2 overexpression induce nuclear localization of Nrf2 and HO-1 expression. **A**: Western blot analysis of Nrf2 in HEK293T cells using anti-c-Myc antibody. **B**: Western blot analysis of Nrf2 in H9c2 cells. Upper band is nuclear Nrf2 (N-Nrf2) and lower band is cytoplasmic Nrf2 (C-Nrf2). **C**: Quantification of the density of expression level of nuclear Nrf2. n = 3. The data are expressed as mean ± SEM. **D**: Western blot analysis of Nrf2 and HO-1 in Nrf2 overexpressed H9c2 cells. The number means intensity of nuclear Nrf2 (arrows). **E**: Quantification of the density of expression level of nuclear Nrf2. n = 3. The data are expressed as mean ± SEM. *P < 0.05.

To examine the effects of Nrf2 on expression of antioxidants, we used Kozak-linked Nrf2 construct. Nrf2 construct was transfected in HEK293T cells, and exogenous Nrf2 protein was detected using anti-c-Myc antibody. (Fig 5A, S2A Fig). In Fig 2F, we showed that molecular weight of nuclear Nrf2 was higher than that of cytoplasmic Nrf2 in H9c2 cells. Therefore, we examined whether overexpression of Nrf2 changed localization of Nrf2. As shown in Fig 5B and 5C, overexpression of Nrf2 upregulated nuclear Nrf2 (N-Nrf2) expression in normal condition (S2B and S2C Fig). Furthermore, propofol increased the expression of nuclear Nrf2 under conditions of oxidative stress in Nrf2 overexpressed H9c2 cells (Fig 5D and 5E, S2D and S2F Fig). Interestingly, HO-1 was also induced by propofol in Nrf2 overexpressed H9c2 cells (Fig 5D, S2E Fig). These results suggest that overexpression of Nrf2 induced HO-1 expression by promoting nuclear localization of Nrf2 under oxidative stress conditions.

### Effects of propofol on myocardial ischemia-reperfusion injury

**Fig 6:**
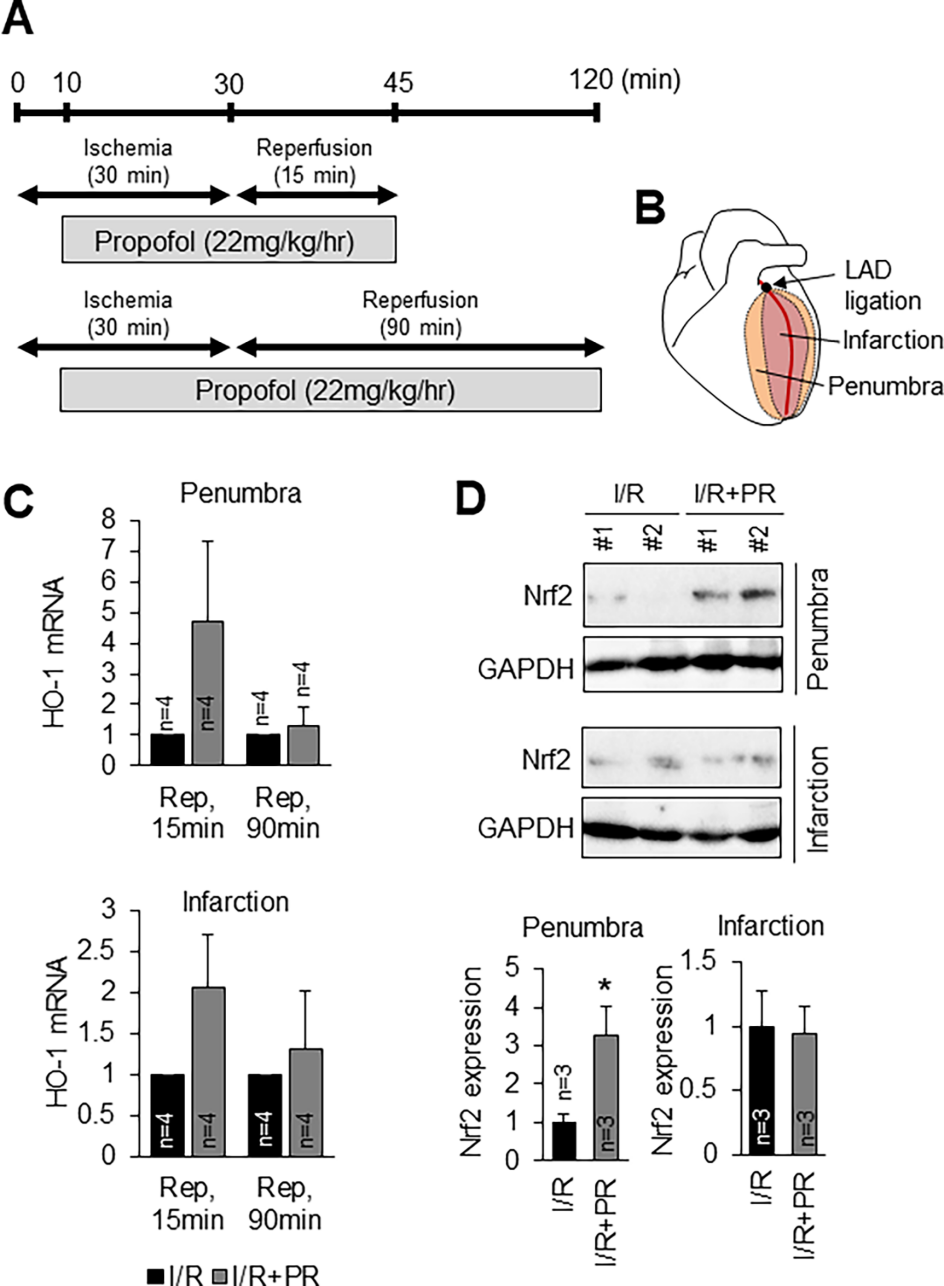
Effects of propofol on myocardial ischemia-reperfusion injury. **A-B**: Scheme of ischemia-reperfusion injury and propofol administration. **C**: Expression profiles of HO-1 mRNA in penumbra and infarction region of ischemia-reperfusion (I/R) group rats and I/R+propofol group rats at 15 min or 90 min after reperfusion. Rep, reperfusion. n = 4. The data are expressed as mean ± SEM. **D**: Western blot analysis of Nrf2 in penumbra and infarction region of I/R group rats and I/R+propofol group rats at 15 min after reperfusion. n = 3. The data are expressed as mean ± SEM.

It is known that propofol protects myocardium against myocardial ischemia-reperfusion (I/R) injury in rat heart model [19]. However, the mechanisms underlying its cardioprotective role remain elusive. Our in vitro studies showed that propofol-induced Nrf2 is critical for cytoprotection against oxidative stress. Therefore, we examined the expression level of Nrf2 downstream enzyme, HO-1 in I/R injury model. The myocardial I/R model was induced by ligation of the left anterior descending (LAD) coronary artery for 30 minutes followed by reperfusion for 15 min or 90 min (Fig 6A and 6B). In the I/R+propofol group, 22mg/kg/hr propofol was administered for 35 min or 110 min (Fig 6A). HO-1 mRNA level was increased after 15 min on reperfusion in the I/R+propofol group compared with I/R group in penumbra and infarction region, although the difference did not reach statistical significance (Fig 6C). HO-1 mRNA level was not increased after 90 min on reperfusion in the I/R+propofol group in penumbra and infarction region. We focused on the early stage since HO-1 mRNA tended to be higher at 15 min after reperfusion in propofol-treated rats. We performed Western blotting using ischemia/reperfusion (I/R) group rats and I/R+propofol group rats at 15 min after reperfusion. The Nrf2 level in penumbra region was higher in the I/R+propofol group compared with I/R group in penumbra region (Fig 6D, S3 Fig 3.3-fold of I/R group). In infarction region, Nrf2 expression level was not changed between I/R+propofol group and I/R group (Fig 6D, S3 Fig). These results suggest that propofol increased the Nrf2 and its downstream enzyme especially in penumbra region at the early stage of reperfusion.

## Discussion

Propofol has been proposed to contribute to the protection of cells or tissue against oxidative stress [5, 6]. However, the underlying mechanism of this beneficial effect is not clear. It is widely accepted that mitochondria play a key role in the development of oxidative stress, and the major endogenous sources of ROS are localized to mitochondria. In the present study, we used exogenous H_2_O_2_ to investigate directly the role of propofol against ROS. As shown in Fig 1, propofol inhibits H_2_O_2_-induced apoptosis of H9c2 cells. Antioxidants such as HO-1 and Nqo-1 are important components of the cellular defense mechanism against oxidative stress [10]. We showed that propofol and H_2_O_2_ co-treatment increased the expression of HO-1 by promoting Nrf2 nuclear localization in H9c2 cells. In other words, propofol has protective effects only if cells are under oxidative stress. Interestingly, we found that the mRNA expression level of Nrf2 was increased by propofol in the presence of H_2_O_2_ (S1J Fig). Propofol appears to regulate Nrf2 expression at the transcription level; however, propofol treatment alone did not increase Nrf2 mRNA expression. These results indicate that propofol and H_2_O_2_ synergistically increase Nrf2 mRNA expression in H9c2 cells. Identification of the molecular mechanisms that regulate Nrf2 transcription will be the next step.

Interestingly, propofol (100 μM) did not protect 1000 μM H_2_O_2_-treated H9c2 cells from cell death (data not shown), suggesting that extreme conditions may preclude the protective effects of propofol. We also observed that 1000 μM propofol, but not 100 μM, caused cell death in H9c2 cells (Fig 1B and 1C). It has been reported that excessive propofol treatment for a prolonged period causes injury to multiple cell types [9, 18]. Indeed, abuse of propofol treatment causes severe complications in patients with critical illness and is called propofol infusion syndrome [20]. The mechanism of cellular cytotoxicity caused by propofol overdose is still unclear. Our aim was to investigate protective roles of propofol against ROS, and we therefore used 100 μM propofol in this study.

Nrf2 activates transcription of many cytoprotective enzymes [21], and HO-1, Nqo-1, gluthathione S-transferase and superoxide dismutase are representative antioxidant enzymes whose expression is regulated by Nrf2 [10, 13, 14, 21]. The activation mechanism of Nrf2 has been studied extensively. Hao et al. reported that polyphenolic compound, resveratrol alleviates endotoxin-induced myocardial toxicity via the Nrf2 transcription factor [15]. In addition, Ryu et al. reported that 7, 8-dihydroxyflavone protects human keratinocytes against oxidative stress-induced cell damage via the ERK and PI3K/Akt-mediated Nrf2/HO-1 signaling pathways [22]. To examine the importance of Nrf2 induced by propofol, we disrupted Nrf2 expression using siRNA. Nrf2, Nqo-1 and HO-1 mRNA levels were higher in cells treated with H_2_O_2_ or H_2_O_2_/propofol than in non-treated cells due to incomplete knockdown by Nrf2 siRNA. Nonetheless, Nqo-1 mRNA was significantly reduced by Nrf2 siRNA (Fig 3), but not by siRNA against HO-1 (data not shown). In addition, propofol did not reduce the H_2_O_2_-induced cytotoxicity of H9c2 cells transfected with Nrf2 siRNA (Fig 4). When we conducted the same experiment using HO-1 or Nqo-1 siRNA, propofol reduced the H_2_O_2_-induced cytotoxicity of H9c2 cells to the same extent as scramble siRNA (Fig 4C and 4D). In addition, to examine the effects of Nrf2 on expression of antioxidants and cytoprotection, we used Kozak-linked Nrf2 construct. Exogenous Nrf2 protein was detected using anti-c-Myc antibody. (Fig 5). However, Western blot analysis showed that a band of Nrf2 protein in cell lysates was weak. It has been reported that under normal conditions, Nrf2 and Keap1 are complexed in the cytoplasm where they are targeted for degradation [10]. The exogenous Nrf2 protein might be degraded in our in vitro assay system. Indeed, propofol did not reduced the H_2_O_2_-induced cytotoxicity of H9c2 cells transfected with Nrf2 construct (H_2_O_2_, 33.4% ± 3.0%; H_2_O_2_+PR, 32.0% ± 2.7%) (S2G Fig). However, overexpression of Nrf2 upregulated nuclear localization of Nrf2 even a band of Nrf2 protein in cell lysates was weak (Fig 5). Furthermore, HO-1 was also induced by propofol in Nrf2 overexpressed H9c2 cells (Fig 5). These results suggested that overexpression of Nrf2 induced HO-1 expression by promoting nuclear localization of Nrf2 under oxidative stress conditions. These results suggest that propofol-induced Nrf2 expression is critical for antioxidant expression.

It is known that propofol protects myocardium against myocardial ischemia-reperfusion (I/R) injury in the rat heart model [19]. However, the mechanisms underlying its cardioprotective role remain elusive. We examined expression levels of Nrf2 in ischemia-reperfusion injury model. Nrf2 was increased after 15 min on reperfusion in the I/R+propofol group compared with I/R group in penumbra and infarction region (Fig 6D). In our study, the myocardial I/R model was induced by ligation of the LAD coronary artery for 30 minutes followed by reperfusion for 15 min or 90 min. HO-1 and Nrf2 were increased after 15 min on reperfusion in propofol-treated rats (Fig 6C and 6D). These results suggest that propofol-induced Nrf2/HO-1 expression is increased in the early stage of reperfusion at penumbra region. Wang et al. reported that administration of propofol ameliorated the cardiac function of rats at 2 h after reperfusion [23]. It is presumed that the expression of Nrf2 and its downstream enzyme including HO-1 are elevated in the early reperfusion stage and contribute to restoration of cardiac function.

In summary, we showed that propofol increased cell survival and reduced H_2_O_2_-induced apoptosis in rat cardiac H9c2 cells. We also showed that propofol promotes nuclear localization of Nrf2 under conditions of oxidative stress. These results suggest that propofol enhances nuclear Nrf2 accumulation and the expression of its downstream enzyme(s), thereby protecting cardiac H9c2 cells from oxidative stress-induced cell death. Further characterization of propofol and investigation of the mechanism by which it regulates gene expression should provide new insights into its crucial role under oxidative stress conditions.

## Supporting information

S1 Fig Propofol and H_2_O_2_ synergistically increase HO-1 expression and upregulate nuclear localization of Nrf2 in H9c2 cells. **A-I**: Full uncropped Western blot images corresponding to Fig 2. J: mRNA expression profile for Nrf2. n = 3. The data are expressed as mean ± SEM.
(TIF)

S2 Fig Nrf2 overexpression induce nuclear localization of Nrf2 and HO-1 expression. **A-F**: Full uncropped Western blot images corresponding to Fig 5. **G**: Effect of exogenous Nrf2 protein on propofol-induced cytoprotection after at 24 h after treatment with H_2_O_2_. Cytotoxicity was measured using an LDH assay. n = 3. The data are expressed as mean ± SEM.
(TIF)

S3 Fig Effects of propofol on myocardial ischemia-reperfusion injury. Full uncropped Western blot images corresponding to Fig 6.
(TIF)
