# Supplementary figures and images for: Propofol induces nuclear localization of Nrf2 under conditions of oxidative stress in cardiac H9c2 cells
Source: PLoS One. 2018 Apr 24;13(4):e0196191. doi: 10.1371/journal.pone.0196191 (PMC5915683; doi:10.1371/journal.pone.0196191)

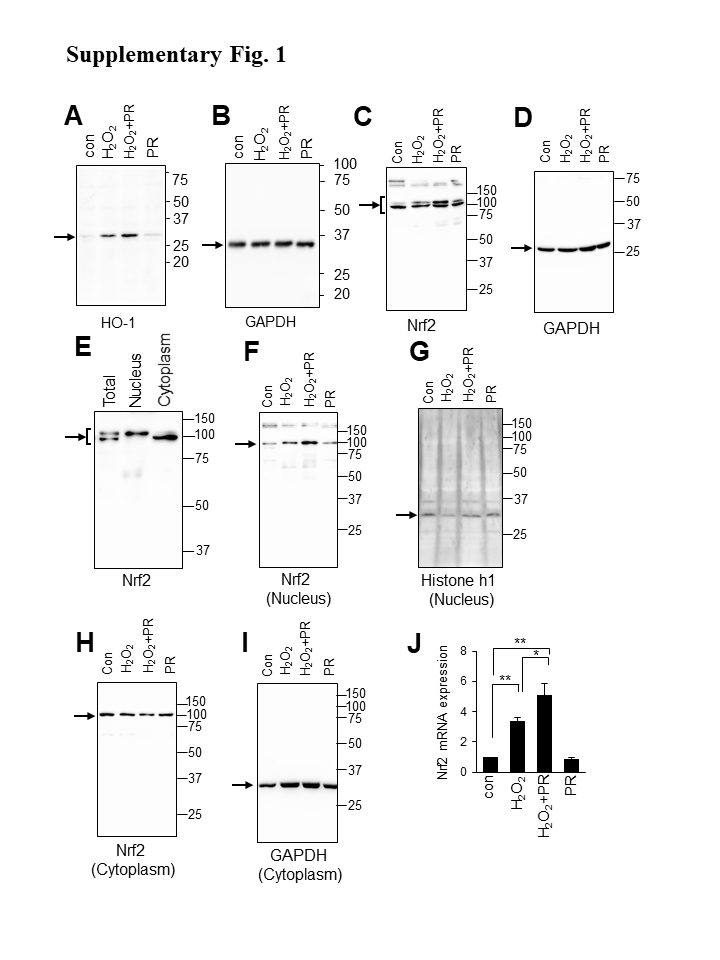

Supplement: S1 Fig — A-I: Full uncropped Western blot images corresponding to Fig 2. J: mRNA expression profile for Nrf2. n = 3. The data are expressed as mean ± SEM. (TIF) [file pone.0196191.s001.tif]

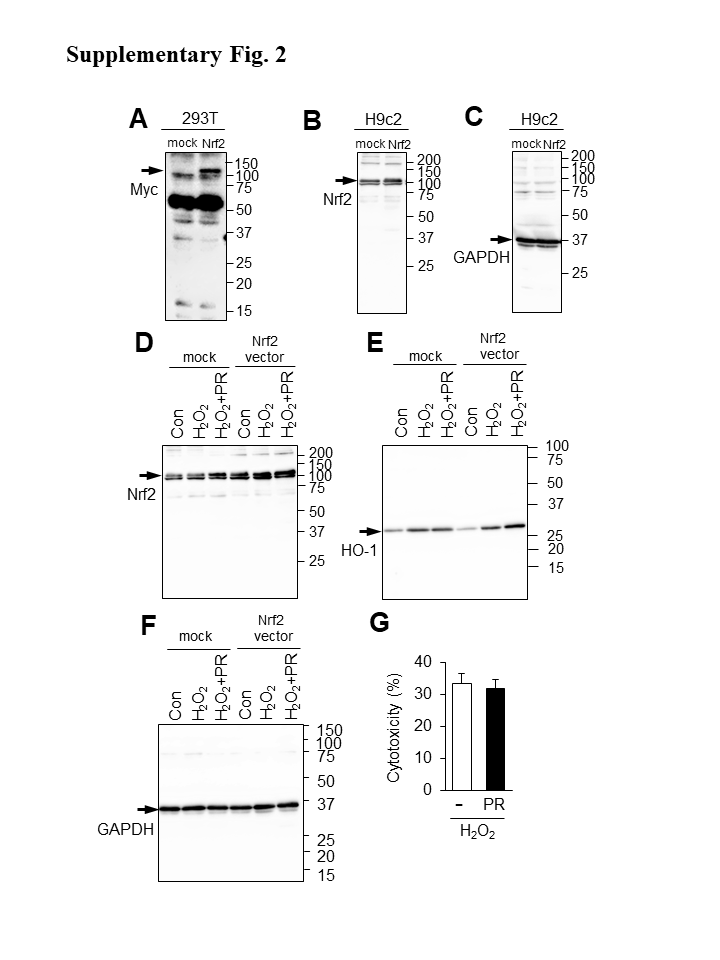

Supplement: S2 Fig — A-F: Full uncropped Western blot images corresponding to Fig 5. G: Effect of exogenous Nrf2 protein on propofol-induced cytoprotection after at 24 h after treatment with H2O2. Cytotoxicity was measured using an LDH assay. n = 3. The data are expressed as mean ± SEM. (TIF) [file pone.0196191.s002.tif]

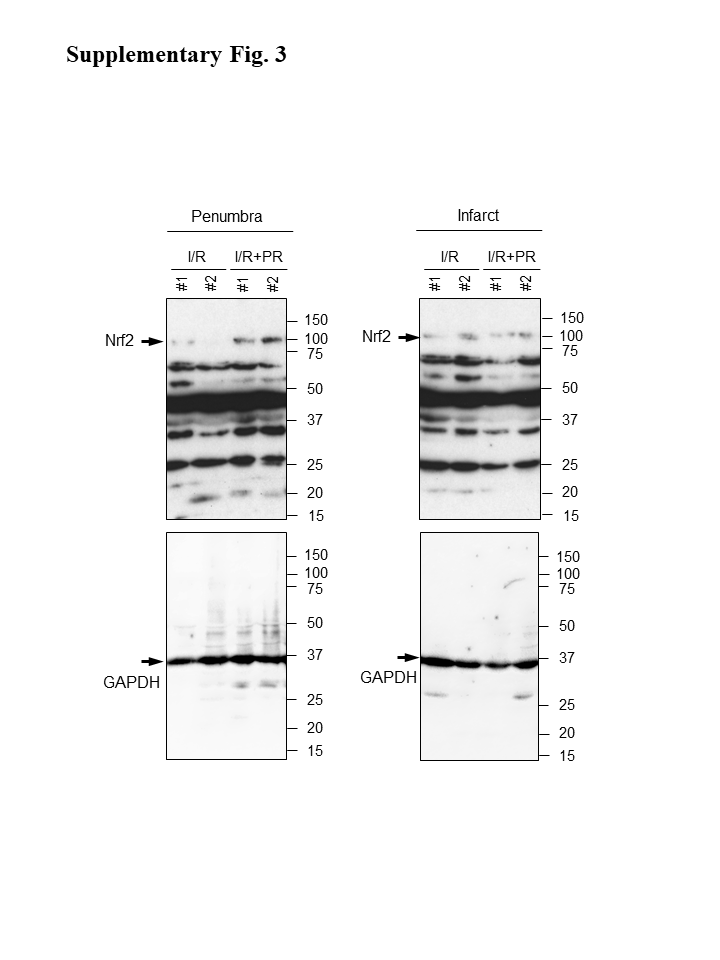

Supplement: S3 Fig — Full uncropped Western blot images corresponding to Fig 6. (TIF) [file pone.0196191.s003.tif]
